# Gender Prediction for a Multiethnic Population via Deep Learning Across Different Retinal Fundus Photograph Fields: Retrospective Cross-sectional Study

**DOI:** 10.2196/25165

**Published:** 2021-08-17

**Authors:** Bjorn Kaijun Betzler, Henrik Hee Seung Yang, Sahil Thakur, Marco Yu, Ten Cheer Quek, Zhi Da Soh, Geunyoung Lee, Yih-Chung Tham, Tien Yin Wong, Tyler Hyungtaek Rim, Ching-Yu Cheng

**Affiliations:** 1 Yong Loo Lin School of Medicine National University of Singapore Singapore Singapore; 2 Ophthalmology and Visual Science Academic Clinical Program Duke-NUS Medical School Singapore Singapore; 3 Singapore Eye Research Institute Singapore Singapore; 4 Medi Whale Inc Seoul Republic of Korea

**Keywords:** deep learning, artificial intelligence, retina, gender, ophthalmology

## Abstract

**Background:**

Deep learning algorithms have been built for the detection of systemic and eye diseases based on fundus photographs. The retina possesses features that can be affected by gender differences, and the extent to which these features are captured via photography differs depending on the retinal image field.

**Objective:**

We aimed to compare deep learning algorithms’ performance in predicting gender based on different fields of fundus photographs (optic disc–centered, macula-centered, and peripheral fields).

**Methods:**

This retrospective cross-sectional study included 172,170 fundus photographs of 9956 adults aged ≥40 years from the Singapore Epidemiology of Eye Diseases Study. Optic disc–centered, macula-centered, and peripheral field fundus images were included in this study as input data for a deep learning model for gender prediction. Performance was estimated at the individual level and image level. Receiver operating characteristic curves for binary classification were calculated.

**Results:**

The deep learning algorithms predicted gender with an area under the receiver operating characteristic curve (AUC) of 0.94 at the individual level and an AUC of 0.87 at the image level. Across the three image field types, the best performance was seen when using optic disc–centered field images (younger subgroups: AUC=0.91; older subgroups: AUC=0.86), and algorithms that used peripheral field images had the lowest performance (younger subgroups: AUC=0.85; older subgroups: AUC=0.76). Across the three ethnic subgroups, algorithm performance was lowest in the Indian subgroup (AUC=0.88) compared to that in the Malay (AUC=0.91) and Chinese (AUC=0.91) subgroups when the algorithms were tested on optic disc–centered images. Algorithms’ performance in gender prediction at the image level was better in younger subgroups (aged <65 years; AUC=0.89) than in older subgroups (aged ≥65 years; AUC=0.82).

**Conclusions:**

We confirmed that gender among the Asian population can be predicted with fundus photographs by using deep learning, and our algorithms’ performance in terms of gender prediction differed according to the field of fundus photographs, age subgroups, and ethnic groups. Our work provides a further understanding of using deep learning models for the prediction of gender-related diseases. Further validation of our findings is still needed.

## Introduction

An individual’s gender is associated with a variety of systemic and ocular diseases. Females have longer life expectancies compared to those of males, regardless of their educational, economic, political, and health statuses [1,2]. Decreased estrogen production predisposes postmenopausal women to degenerative conditions, including cataracts and age-related macular degeneration [3-8]. In contrast, males are predisposed to open-angle glaucoma [9], diabetic retinopathy [10], and pigment dispersion glaucoma [11].

Deep learning algorithms have been developed for the detection of systemic and eye diseases based on fundus photographs [12-21]. By using deep neural networks, Poplin et al [12] found that cardiovascular risk factors, including gender, can be predicted with fundus images and obtained good classification results with a data set comprising White individuals. More recently, Gerrits et al [17] and Kim et al [22] also predicted gender by using neural networks to analyze Qatari and South Korean data sets, respectively.

This study builds on preexisting literature in three ways. First, we predicted gender by using retinal fundus images from a Southeast Asian data set. Second, we evaluated how differing fundus photography fields could have an effect, if any, on gender classification results. This is worth exploring because the retina possesses features that can be affected by gender differences (eg, vessel structure; optic nerve, fovea, and macular morphology; and retinal pigmentation). Different fundus photography fields (optic disc–centered, macula-centered, and peripheral fields) capture these features to varying extents and affect these features’ availability in a neural network. Rim et al [22] reported the good generalizability of similar deep learning algorithms that have been used to predict gender based on fundus photographs; however, intracohort subgroup comparisons were not performed. Understanding how model performance differs based on different ethnic, age, and image field subgroups will be useful [22].

Third, the diversity of our data set allowed for the comparison of algorithm performance across age and ethnic subgroups (Malay, Chinese, and Indian subgroups). The introduction of artificial intelligence in clinical medicine has brought about ethical concerns, of which one is problematic decision-making by algorithms that reflect biases that are inherent in the data used to train these algorithms [23]. Ensuring that our model generalizes well across different ethnicities is essential for avoiding inadvertent, subtle discrimination in health care delivery [24]. Cross-cultural analysis is a unique feature of our study—one that is lacking in existing literature on deep learning in ophthalmology because few populations are inherently diverse.

## Methods

### Ethics Statement

This retrospective cross-sectional study was approved by the institutional ethical committee and adhered to the tenets of the Declaration of Helsinki. The need to obtain written informed consent was waived due to the use of anonymized and deidentified data.

### Study Population

The Singapore Epidemiology of Eye Diseases (SEED) study is a population-based study that recruited subjects from the three major ethnic groups (the Chinese, Malay, and Indian ethnic groups) in Singapore. The SEED study’s baseline examinations were conducted from 2004 through 2011, and subsequent follow-up studies were performed, as follows: the Singapore Malay Eye Study (baseline examination: 2004-2006; follow-up examination: 2010-2013), the Singapore Indian Eye Study (baseline examination: 2007-2009; follow-up examination: 2013-2016), and the Singapore Chinese Eye Study (baseline examination: 2009-2011; follow-up examination: 2016-2018). The detailed methodology of the SEED study was published previously [25-28]. Briefly, an age-stratified random sampling method was used to select subjects aged ≥40 years from each ethnic group living across southwestern Singapore. In total, 3280 out of 4168 Malay individuals (78.7%), 3400 out of 4497 Indian individuals (75.6%), and 3353 out of 4606 Chinese individuals (72.8%) agreed to participate in the study. As such, an overall response rate of 75.6% was achieved. The entire data set, which included both visits, was split and used for algorithm development and testing.

### Fundus Photography and Image Database

A digital, nonmydriatic retinal camera (Canon CR-1 Mark-II nonmydriatic, digital retinal camera; Canon Inc) was used to obtain fundus photographs according to Early Treatment for Diabetic Retinopathy Study (ETDRS) standard fields 1 to 5. This was done after performing pharmacological dilation with 1% tropicamide and 2.5% phenylephrine hydrochloride. A total of 175,038 fundus photographs from 10,033 SEED study participants were included in this study. Original fundus photographs (3504×2336 pixels) were extracted in the JPEG format, and the black space around the contours of each photograph was removed. All images were reformatted to 300×300-pixel images.

### Model Development

**Figure 1:**
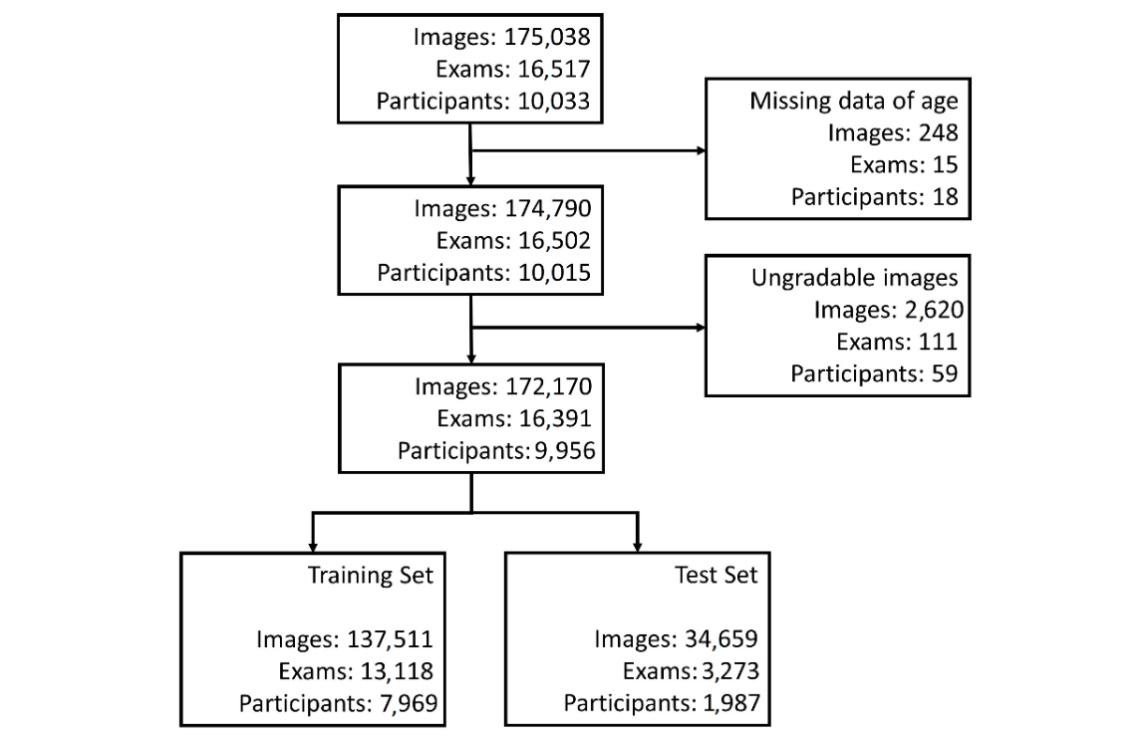
Flowchart depicting the inclusion and exclusion of study images and participants.

Separate models for 3 different focus fields of fundus photographs were developed (optic disc–centered, macula-centered, peripheral fields) [29]. Images without age and gender information or those deemed ungradable were excluded from the analysis. The gradeability of fundus photographs was manually determined based on a modification of the Wisconsin Age-Related Maculopathy Grading System [30]. A total of 172,170 fundus photographs (from the 16,391 examinations of 9956 participants) were divided into a training set (137,511/172,170, 79.9%) for developing our models and a test set (34,659/172,170, 20.1%), which was reserved to evaluate model performance. The photographs were stratified according to age groups, gender, and ethnic groups. Figure 1 and Table 1 describe this split in more detail. The test set was not used during model development. This division of photographs was based on the individual level rather than the image level to avoid class imbalances. Dividing photographs at the individual level ensured that there was an equal number of images for each individual, thereby avoiding the potential skew of data. Data augmentation (random rotation from −5 to 5 degrees and random brightness adjustment) was performed to introduce invariance in our neural network [31,32].

**Table 1:** Population characteristics.

Characteristics				Training set, n (%)		Test set, n (%)		*P* value	
**Fundus photographs (N=175,038)^a^**									
	Optic disc–centered photographs		56,814 (41.3)		14,231 (41.1)		.45		
	Macula-centered photographs		53,863 (39.2)		13,705 (39.5)		N/A^b^		
	Other peripheral photographs		26,834 (19.5)		6723 (19.4)		N/A		
**Examinations (N=16,517)^c^**									
	**Age group (years)**								.75
		40-49	2145 (16.4)		551 (16.8)				
		50-59	4447 (33.9)		1105 (33.8)				
		60-69	3772 (28.8)		915 (28)				
		≥70	2754 (21)		702 (21.5)				
	**Gender**								>.99
		Female	6725 (51.3)		1678 (51.3)				
		Male	6393 (48.7)		1595 (48.7)				
	**Ethnic groups**								.80
		Malay	4067 (31)		1024 (31.3)				
		Chinese	4609 (35.1)		1161 (35.5)				
		Indian	4442 (33.9)		1088 (33.2)				

^a^The training set included a total of 137,511 fundus photographs, and the test set included a total of 34,659 fundus photographs.

^b^N/A: not applicable.

^c^The training set included data on a total of 13,118 examinations, and the test set included data on a total of 3273 examinations.

Our deep learning model, which was based on the Visual Geometry Group-16 neural network architecture [33], was developed, trained, and evaluated in TensorFlow [34,35]. The model had 13 convolutional layers after batch normalization and a fully connected layer after compressing the feature vector via global average pooling. The Adam optimizer with fixed weight decay was used to train our model; the learning rate was set to 0.0001 for 100 epochs. At the end of the neural network, a prediction score was generated for binary classification. A low prediction score was classified as “male,” while a high prediction score was classified as “female.” With regard to model explanation, saliency maps created via guided gradient-weighted class activation mapping (Grad-CAM) [36,37] were superimposed over input images to facilitate our understanding of how our model predicted gender.

### Reference Standard

Gender information (male or female) was collected from the SEED study participants’ National Registration Identity Card, which is provided to all Singapore citizens.

### Subgroups

Age was calculated based on the birth date indicated on participants’ National Registration Identity Card. The younger subgroup included participants aged 40 to 65 years, while the older subgroup included those aged ≥65 years. To classify the three ethnic subgroups, our study used criteria that were set by the Singapore census to define *Malay*, *Chinese*, and *Indian* [25,27].

### Statistical Analysis

Python packages, including NumPy, SciPy, matplotlib, scikit-learn, were used to process the data [38]. Performance was evaluated by using the internal validation set, which included 34,659 fundus photographs (14,231 optic disc–centered field images, 13,705 macula-centered field images, and 6723 peripheral field images). Receiver operating characteristic curves for binary classification were plotted. The DeLong test for area under the receiver operating curve (AUC) comparisons was used [39]. Individual-based and image-based analyses were conducted.

## Results

A total of 172,170 fundus photographs, including 71,045 optic disc–centered field images, 67,568 macula-centered field images, and 33,557 peripheral field images, were distributed among the training and test sets (Table 1). The mean age of participants was 60.8 years (SD 10.3 years; minimum: 40.0 years; maximum: 91.3 years), and 48.7% (7988/16,391) of the participants were male. The distribution of photographs between the training and test sets was stratified according to gender, age subgroups, and the three ethnic subgroups.

**Figure 2:**
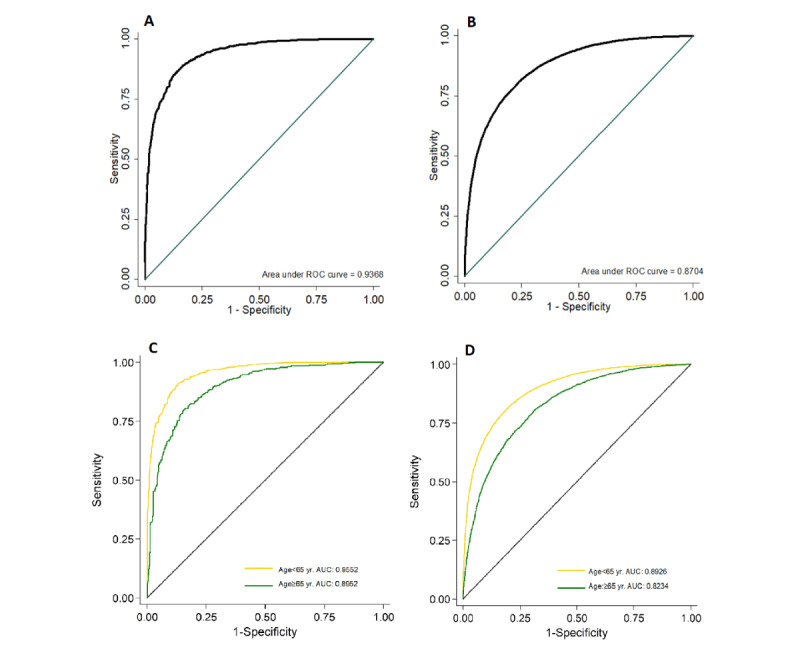
ROC curves at the individual and image levels based on the internal test set. A: Individual level; total population. B: Image level; total images. C: Individual level; age subgroups. D: Image level; age subgroups. Upon internal testing, the AUCs achieved were 0.937 and 0.870 at the individual and image levels (A and B), respectively. The AUCs achieved in the younger subgroups (aged <65 years) were 0.955 and 0.893 at the individual and image levels, respectively (*P*<.001). The AUCs achieved for the older subgroups were 0.895 and 0.823 at the individual and images levels, respectively (*P*<.001). AUC: area under the receiver operating curve; ROC: receiver operating curve.

Upon validation, the model achieved an AUC of 0.94 (95% CI 0.93-0.95) at the individual level and an AUC of 0.87 (95% CI 0.87-0.87) at the image level (Figure 2). With regard to the age subgroup analysis at the individual level, model performance was better in the younger group (aged 40-65 years; AUC=0.96; 95% CI 0.95-0.96) than in the older group (aged >65 years; AUC=0.90; 95% CI 0.88-0.91; *P<*.001). At the image level, model performance in the younger group also surpassed model performance in the older group; AUCs of 0.89 (95% CI 0.89-0.90) and 0.82 (95% CI 0.82-0.83), respectively, were achieved (*P*<.001).

**Figure 3:**
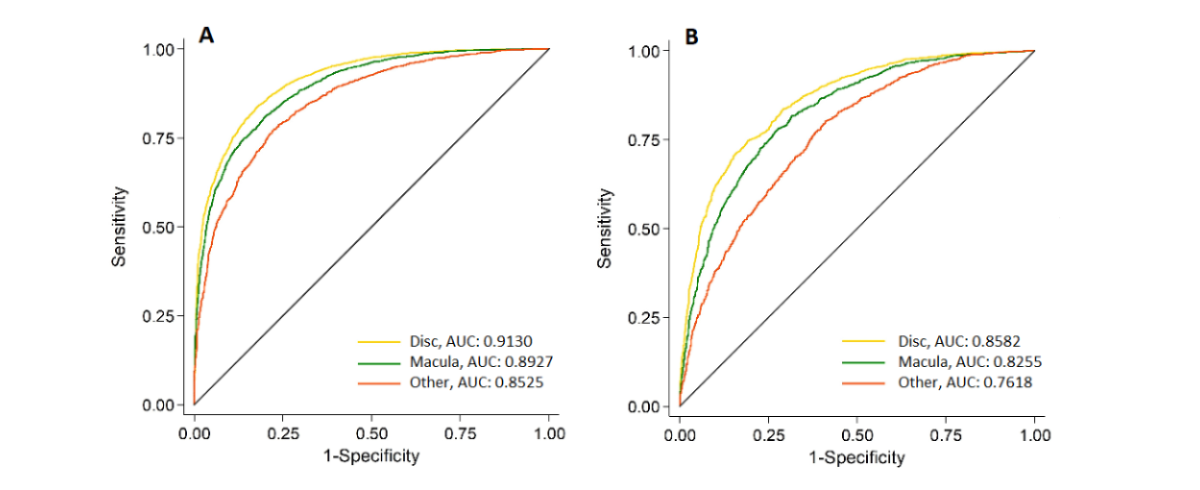
Comparison of the algorithms’ performance in gender prediction between the different fundus photograph fields (optic disc–centered, macula-centered, and peripheral or other fields) A: Age<65 years. B: Age≥65 years. AUC: area under the receiver operating curve.

We examined the differences in the model’s predictions of gender across the three fundus photography fields at the image level. Figure 3 describes the corresponding AUC curves. The model’s overall performance was better in the younger group (Figure 3) than in the older group (Figure 3). In both age groups, optic disc–centered images resulted in the best performance in terms of gender prediction. In the younger age group, the AUC was 0.91 (95% CI 0.91-0.92) for the optic disc–centered images, 0.89 (95% CI 0.89-0.90) for the macula-centered images, and 0.85 (95% CI 0.84-0.86) for the peripheral field images (*P*<.001). In the older age group, the AUC was 0.86 (95% CI 0.85-0.87) for the optic disc–centered images, 0.83 (95% CI 0.81-0.84) for the macula-centered images, and 0.76 (95% CI 0.84-0.86) for the peripheral field images (*P*<.001).

**Figure 4:**
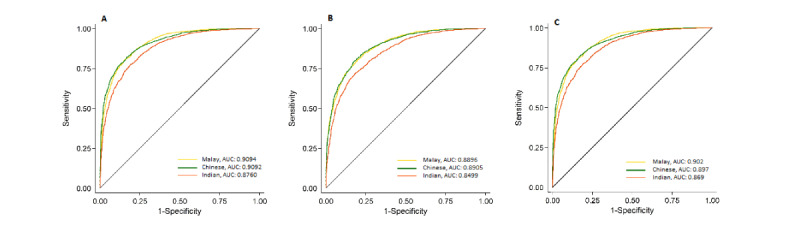
Comparison of the algorithms’ performance in gender prediction between ethnic groups. A: Optic disc–centered photographs. B: Macula-centered photographs. C: Overall. AUC: area under the receiver operating curve.

We also evaluated the model’s gender prediction performance according to ethnic groups (the Malay, Chinese, Indian groups). Figure 4 depicts our algorithms’ performance in analyzing photographs at the image level; the model fared relatively well for the Malay and Chinese ethnic groups but fared suboptimally for the Indian ethnic group. The model’s overall performance was better when using optic disc–centered images (Figure 4) than when using macula-centered images (Figure 4). With regard to the optic disc–centered image group, the AUC was 0.91 (95% CI 0.90-0.92) for the Malay group, 0.91 (95% CI 0.90-0.92) for the Chinese group, and 0.88 (95% CI 0.87-0.89) for the Indian group (*P*<.001). With regard to the macular-centered image group, the AUC was 0.890 (95% CI 0.88-0.90) for the Malay group, 0.89 (95% CI 0.88-0.90) for the Chinese group, and 0.85 (95% CI 0.84-0.86) for the Indian group (*P*<.001). No significant performance differences were observed between the Malay and Chinese ethnic groups (optic disc–centered images: *P=*.98; macula-centered images: *P*=.90). Precision-recall curves were generated in addition to the receiver operating curves. These are provided in Multimedia Appendix 1.

**Figure 5:**
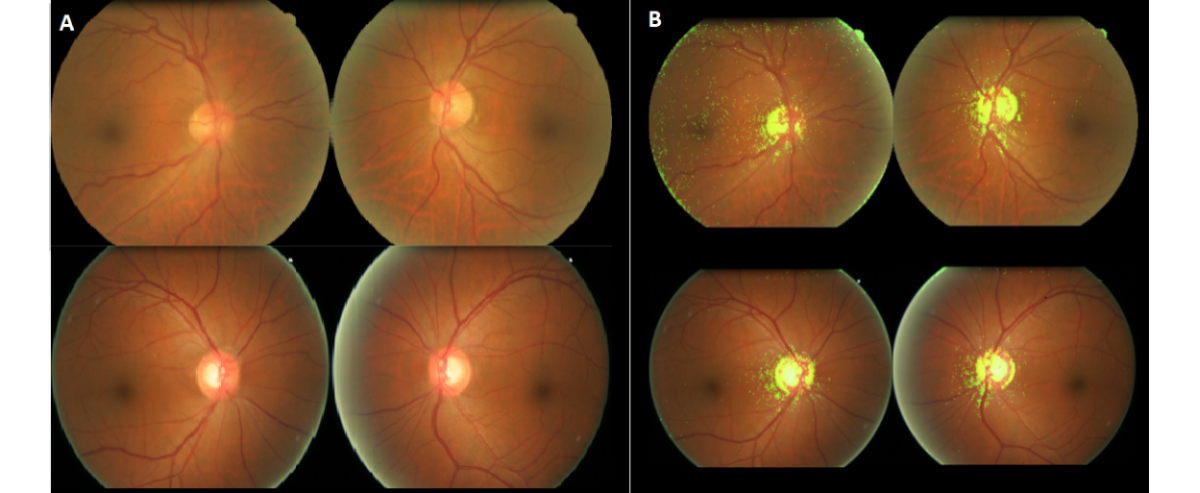
Original fundus photographs (A) and overlaid heat maps (B) with the features that were most associated with the male gender.

**Figure 6:**
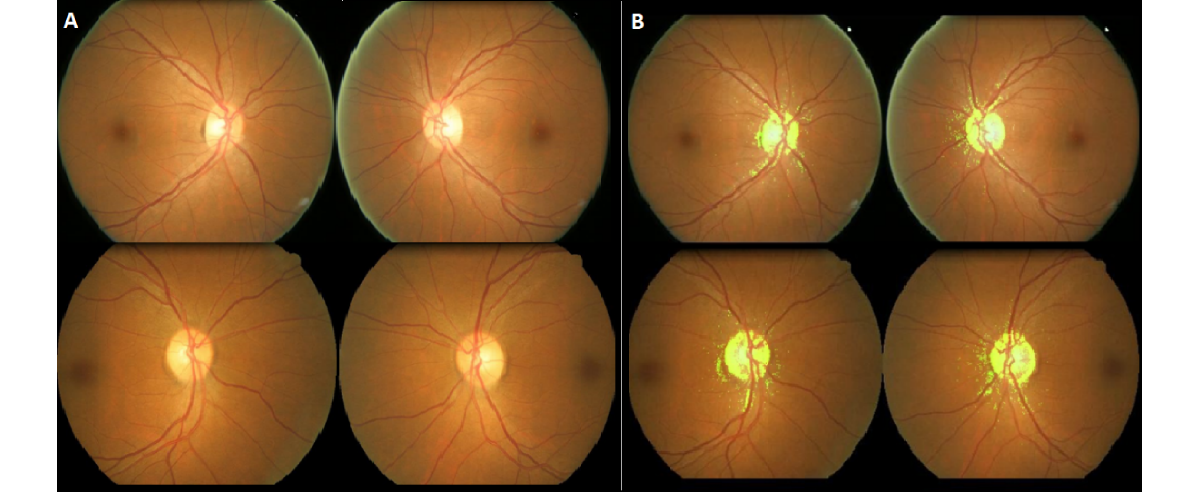
Original fundus photographs (A) and overlaid heat maps (B) with the features that were most associated with the female gender.

**Figure 7:**
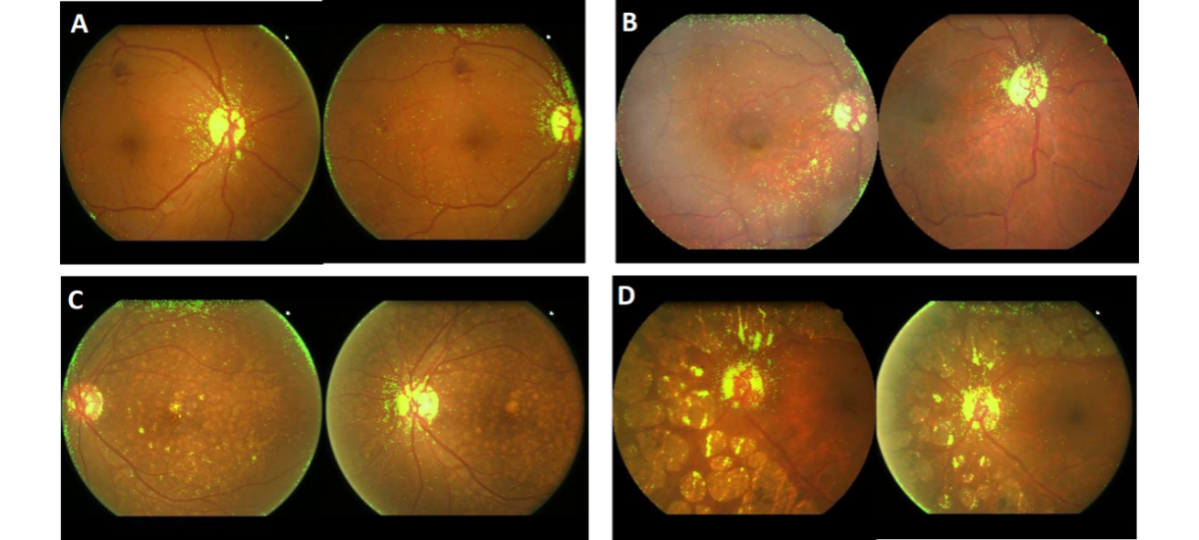
Selected heat maps of fundus images showing pathological lesions (all images are optic disc–centered images). A: Images of diabetic retinopathy. B: Images of hypertensive retinopathy. C: Images of age-related macular degeneration. D: Images of laser scars.

Saliency maps (heat maps) were generated via Grad-CAM for model explanation. Fundus photographs and overlaid heat maps that were strongly associated with males and females (extreme binary classification prediction scores) are shown in Figure 5 and Figure 6, respectively. The optic disc and the surrounding structures are activated in every heat map in Figure 5 and Figure 6. Selected heat maps of fundus images showing pathological lesions are presented in Figure 7. These heat maps suggested that the optic disc was an area of interest in gender prediction, despite the presence of random distractive elements (laser scars, diabetic retinopathy, hypertensive retinopathy, and age-related macular degeneration). A similar trend was noted in the heat maps of macula-centered images.

## Discussion

### Principal Findings

In this study, our results demonstrated the following points: (1) model performance was better in the younger subgroup (aged 40-65 years) than in the older subgroup (aged >65 years); (2) optic disc–centered images provided the most accurate predictions for gender, followed by macula-centered images; (3) the model’s performance was better in the Malay and Chinese ethnic subgroups than in the Indian ethnic subgroup; and (4) the algorithms functioned well in the presence of possibly distractive attributes.

The deep learning algorithm from Poplin and colleagues [12] was developed based on 48,101 and 236,234 color fundus photographs from the UK Biobank and Eye Picture Archive Communication System (EyePACS) data sets, respectively. It successfully predicted gender and achieved an AUC of 0.97 (95% CI 0.97-0.97) and 0.97 (95% CI 0.96-0.98) with the UK Biobank and the EyePACS-2K validation sets, respectively. Compared to the model developed by Poplin and colleagues [12], our model, which achieved an AUC of 0.94 (95% CI 0.93-0.95), is slightly less precise. However, our model was trained on and validated with a wider range of age groups than those of Poplin et al [12], and this could explain the relatively weaker performance of our algorithm; we confirmed that the algorithms’ performance was lower in older subgroups.

The ability of neural networks to use greater abstractions and tighter integrations comes at the cost of lower interpretability [40]. Saliency maps, which are also called *heat maps* or *attention maps*, are common model explanation tools that are used to visualize model thinking by indicating areas of local morphological changes within fundus photographs that carry more weight in modifying network predictions. After using saliency maps, which were created via Grad-CAM [36,37], we believe that our algorithms mainly used the features of the optic disc for gender prediction. This pattern is consistent with the observations made by Poplin et al [12] in 2018. Deep learning models that were trained by using images from the UK Biobank and EyePACS data sets primarily highlighted the optic disc, retinal vessels, and macula when soft attention heat maps were applied, although there appeared to be a weak signal distributed throughout the retina [12]. Given that the Poplin et al [12] study predominantly used data sets of White (UK Biobank) and Hispanic (EyePACS) individuals and our study used a Southeast Asian population (ie, Malay, Chinese, and Indian individuals), our results suggest that gender predictions based on fundus photographs will likely generalize well across different ethnic groups. Additional validations of our models based on other global population data sets would strengthen these findings.

Figure 4 shows representative fundus photographs with the most masculine and feminine features. The heat maps mainly highlighted the optic discs and the surrounding areas. Our algorithms work well even when there are obvious different clinical characteristics, such as retinal hemorrhages, ghost vessels, laser scars, and silicone oil tamponade eye. To further confirm that the optic disc is an area of interest in gender prediction, we performed an explorative analysis on a subset of fundus images that did not capture the optic disc. Of the 6723 peripheral field images from the test set, 649 images had fields that did not encompass the optic disc. The model validation analysis based on these 649 peripheral field images that did not capture the optic disc returned an AUC of 0.69 (95% CI 0.65-0.73). This explorative comparison found that the model’s performance markedly decreased in the absence of features provided by the optic disc. We can therefore suggest with greater certainty that the optic disc is the main structure used by deep learning algorithms for gender prediction. Kim et al [22] explored this concept in a slightly different manner. They reported a decreased AUC when predicting gender by using subsets of artificially inpainted fundus images, in which either the fovea or retinal vessels were erased. Optic disc omission was not described, although their reported heat maps indicated activations in the fovea, optic disc, and retinal vessels [22]. In addition, Korot et al [41] reported poor performance when using images with foveal pathologies and used this finding to suggest that the fovea is an important input region for gender prediction. However, their saliency maps strongly attributed their model’s predictive power to the optic disc. This is similar to the findings of our study. It is likely that both the fovea and optic disc provide critical feature inputs for gender prediction models, but we are unable to comment on their relative importance.

The consideration of clinical applicability is essential when developing a useful deep learning algorithm. In a real-world setting, clinicians often encounter a mixture of fundus photographs with different fields, and it is common to observe the incorrect sorting of fundus photographs within publicly available data sets [42]. Our results showed that the most precise predictions were obtained when using optic disc–centered images as the model input in both the primary and subgroup analyses. Researchers should be aware of the possible performance differences that arise due to using different image fields when predicting gender or gender-related systemic factors; using optic disc–centered images alone or a combination of macula-centered and optic disc–centered images may be the most prudent approach. Based on our model’s suboptimal performance when using peripheral field images, such images are not ideal input data for gender prediction models.

A common ethical concern with regard to decision-making by algorithms is that biases that are inherent in the data used to train these algorithms will manifest during usage [23]. A study of facial recognition software evaluated the performance of three leading recognition systems (those of Microsoft Corporation, IBM Corporation, and Megvii) in a gender classification task based on human skin tones [43]. The results showed that darker-skinned females were the most misclassified group. The study reported error rates of up to 34.7% for this group. However, a maximum error rate of 0.8% was achieved for lighter-skinned males. The implications of this study raised broad questions about the fairness and accountability of artificial intelligence and contributed to the concept of algorithmic accountability [44]. Based on the ethnic subgroup analysis in our study, our model did not perform as well in predicting gender in the Indian ethnic group (AUC=0.88; 95% CI 0.87-0.89) as it did in predicting gender in the Chinese (AUC=0.91; 95% CI 0.90-0.92) and Malay (AUC=0.91; 95% CI 0.90-0.92) ethnic groups (*P*<.001). Given that our results have shown an undesired disparity in performance among the three ethnic groups, efforts will be needed to refine the model so that gender prediction accuracies across different ethnic groups are reasonably on par. Ensuring that our model generalizes well across different ethnicities is essential for avoiding inadvertent, subtle discrimination in health care delivery [24].

A study limitation is that our model was developed and trained with data from a single center; therefore, the model was exposed to the inadvertent incorporation of systemic error. Ideally, an external validation data set that includes photographs that were taken by using the ETDRS standard fields should also be used to evaluate the algorithms. However, photographs that include only 1 field (eg, only macula-centered photographs) cannot be used alone for comparisons because of the systemic error involved. We were unable to find a well-organized data set that included images with different fundus photography fields for external validation. Training the model by using diverse, independent data sets that are captured by using different instruments and come from a variety of populations and clinical settings will also enhance the model’s generalizability [45]. Another limitation is our algorithms’ limited applicability to younger populations, as our study only included images from individuals aged ≥40 years.

### Conclusions

In summary, our study is, to the best of our knowledge, the first to predict gender based on retinal fundus photographs of a Southeast Asian population. The ethnic diversity of our data set allowed us to make intercultural comparisons. The model’s performance was better in the Malay and Chinese subgroups than in the Indian ethnic subgroup, and more work is required to refine the model and avoid an undesired disparity in performance among different ethnic groups. Our analysis of 3 different retinal fields provides evidence that the optic disc is a critical feature that is used by deep learning models for gender prediction. Algorithms that used peripheral field images had the lowest performance, followed by those that used macula-centered photographs. Algorithms that used optic disc–centered photographs had the best performance. Our work provides a further understanding of using deep learning models for the prediction of gender-related diseases, and we recommend using external validation sets to replicate our results.

## Appendix

Multimedia Appendix 1 Precision-recall curves.

## Abbreviations

AUC: area under the receiver operating curve
ETDRS: Early Treatment for Diabetic Retinopathy Study
EyePACS: Eye Picture Archive Communication System
Grad-CAM: gradient-weighted class activation mapping
SEED: Singapore Epidemiology of Eye Diseases
