# Supplementary figures and images for: Gender Prediction for a Multiethnic Population via Deep Learning Across Different Retinal Fundus Photograph Fields: Retrospective Cross-sectional Study
Source: JMIR Med Inform. 2021 Aug 17;9(8):e25165. doi: 10.2196/25165 (PMC8408758; doi:10.2196/25165)

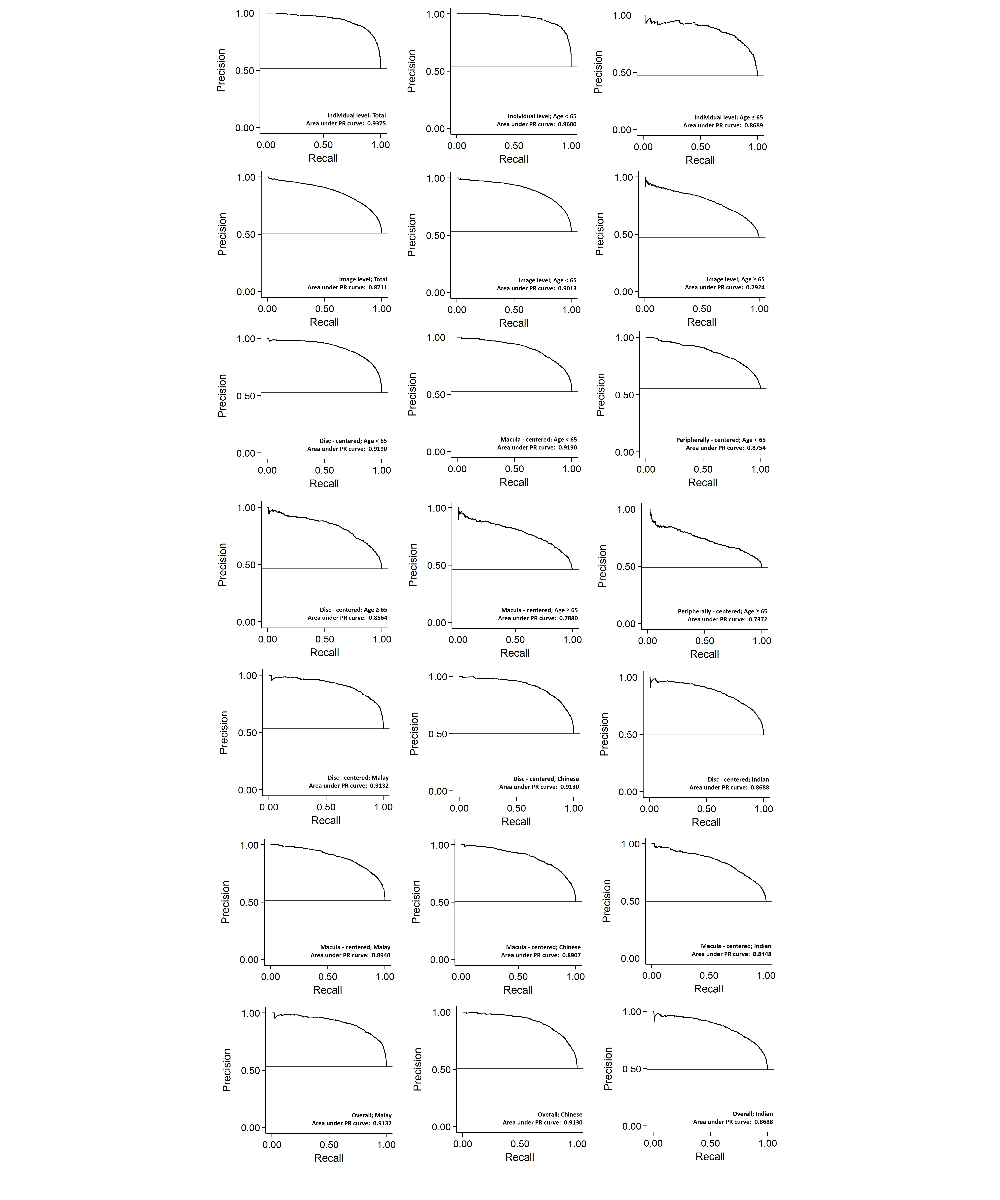

Supplement: Multimedia Appendix 1 [file medinform_v9i8e25165_app1.png]
